# Secreted proteins in treating metabolic dysfunction-associated steatotic liver disease: from bench towards bedside

**DOI:** 10.1093/procel/pwaf027

**Published:** 2025-04-17

**Authors:** Yeping Huang, Bin Liu, Cheng Hu, Yan Lu

**Affiliations:** Shanghai Diabetes Institute, Shanghai Key Laboratory of Diabetes Mellitus, Shanghai Clinical Centre for Diabetes, Shanghai Sixth People’s Hospital Affiliated to Shanghai Jiao Tong University School of Medicine, Shanghai 200233, China; Jiangsu Key Laboratory of Marine Pharmaceutical Compound Screening, College of Pharmacy, Jiangsu Ocean University, Lianyungang 222005, China; Shanghai Diabetes Institute, Shanghai Key Laboratory of Diabetes Mellitus, Shanghai Clinical Centre for Diabetes, Shanghai Sixth People’s Hospital Affiliated to Shanghai Jiao Tong University School of Medicine, Shanghai 200233, China; Institute for Metabolic Disease, Fengxian Central Hospital Affiliated to Southern Medical University, Shanghai 201499, China; Institute of Metabolism and Regenerative Medicine, Digestive Endoscopic Center, Shanghai Sixth People’s Hospital Affiliated to Shanghai Jiao Tong University School of Medicine, Shanghai 200233, China

**Keywords:** secreted proteins, metabolic dysfunction-associated steatotic liver disease, metabolic dysfunction-associated steatohepatitis, hepatic lipid metabolism, clinical application

## Abstract

Metabolic dysfunction-associated steatotic liver disease (MASLD) has become a global epidemic, yet effective pharmacological treatments remain limited. Secreted proteins play diverse roles in regulating glucose and lipid metabolism, and their dysregulation is implicated in the development of various metabolic diseases, including MASLD. Therefore, targeting secreted proteins and modulating associated signaling pathways represents a promising therapeutic strategy for MASLD. In this review, we summarize recent findings on the roles of emerging families of secreted proteins in MASLD and related metabolic disorders. These include the orosomucoid (ORM) family, secreted acidic cysteine rich glycoprotein (SPARC) family, neuregulin (Nrg) family, growth differentiation factor (GDF) family, interleukin (IL) family, fibroblast growth factor (FGF) family, bone morphogenic protein (BMP) family, as well as isthmin-1 (Ism1) and mesencephalic astrocyte-derived neurotrophic factor (MANF). The review highlights their impact on glucose and lipid metabolism and discusses the clinical potential of targeting these secreted proteins as a therapeutic approach for MASLD.

## Introduction

Metabolic dysfunction-associated steatotic liver disease (MASLD), previously known as nonalcoholic fatty liver disease (NAFLD), has become the most common liver disease worldwide (Miao et al., 2024). MASLD encompasses a spectrum of conditions, ranging from simple steatosis to a more serious form known as metabolic dysfunction-associated steatohepatitis (MASH) (Feng et al., 2024; Miao et al., 2024). While the primary cause of simple steatosis is the excessive accumulation of triglycerides (TG) in hepatocytes (Feng et al., 2024; Miao et al., 2024), MASH is characterized by chronic liver inflammation and injury. Moreover, MASH can progress to liver fibrosis, cirrhosis and hepatocellular carcinoma (HCC) (Musale et al., 2023). Alarmingly, global epidemiological data from 2010 to 2019 indicate that MASH is the fastest growing cause of HCC, particularly in the Americas (Huang et al., 2022; Loomba et al., 2021). Mechanistically, the two-hit hypothesis proposed over two decades ago (Day and James, 1998) is now considered outdated. Instead, the multiple-hit hypothesis has gained prominence, incorporating factors such as genetic susceptibility, dysregulation of lipid metabolism in hepatocytes, alterations in gut microbiota, inflammasome activation, and inter-organ interactions (Fabbrini et al., 2010; Friedman et al., 2018; Loomba et al., 2021; Zhao et al., 2023). Currently, Resmetirom, a thyroid hormone receptor β agonist, is the only approved medication for patients with MASLD/MASH (Harrison et al., 2024b; Xie et al., 2024). Although Resmetirom has shown efficacy in achieving MASH resolution, improving fibrosis, and enhancing health-related quality of life, its therapeutic benefits are limited, with only 25%–30% of patients responding to the treatment (Harrison et al., 2024a; Younossi et al., 2025). This highlights the urgent need for more effective therapeutic strategies and underscores the ongoing challenges in developing novel treatments for MASLD/MASH (Tilg et al., 2023).

**Figure 1.**
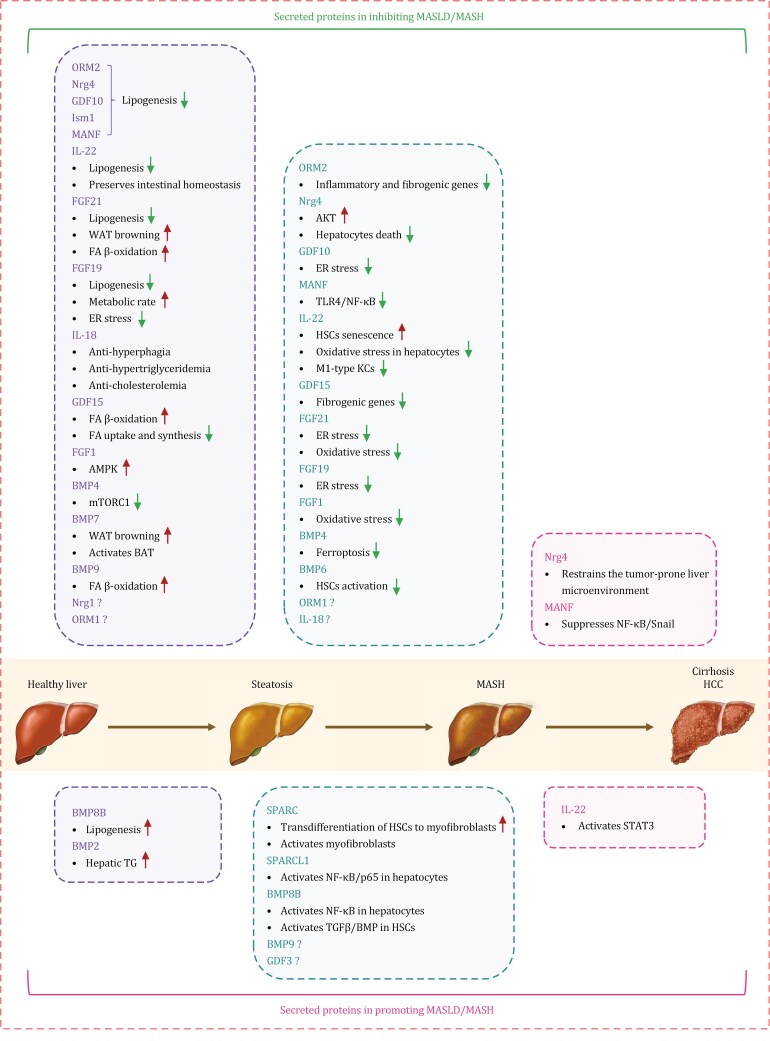
**Roles of secreted proteins in the development and progression of metabolic dysfunction-associated steatotic liver disease.** ORM2, Nrg4, GDF10, Ism1, MANF, IL-22, FGF21, FGF19, IL-18, GDF15, FGF1, BMP4, BMP7, BMP9, Nrg1, and ORM1 can ameliorate hepatic steatosis, while BMP8B and BMP2 facilitate it. ORM2, Nrg4, GDF10, MANF, IL-22, GDF15, FGF21, FGF19, FGF1, BMP4, BMP6, ORM1, and IL-18 can alleviate MASH, whereas SPARC, SPARCL1, BMP8B, BMP9, and GDF3 aggravate it. Nrg4 suppresses the progression from MASH to hepatocellular carcinoma, whereas MANF inhibits hepatocellular carcinoma development. BAT, brown adipose tissue; ER, endoplasmic reticulum; FA, fatty acid; HSCs, hepatic stellate cells; KCs, Kupffer cells; WAT, white adipose tissue.

**Figure 2.**
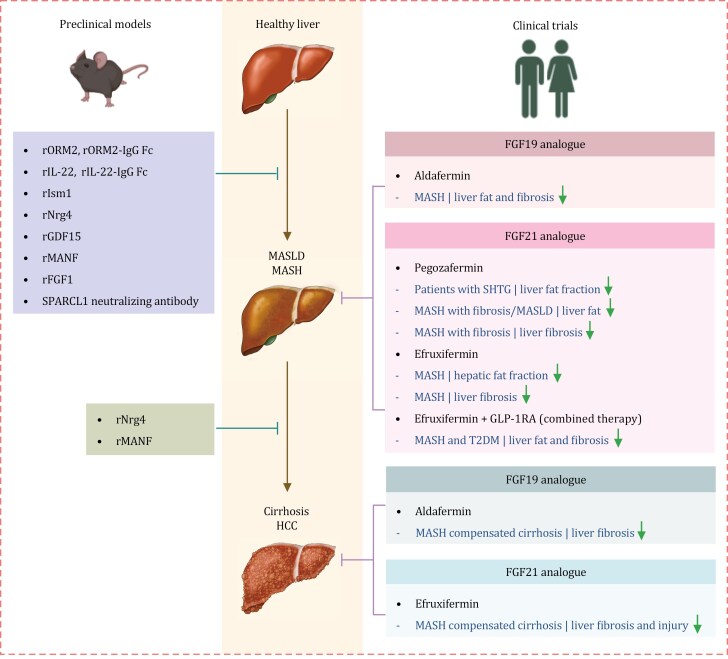
**Secreted proteins as therapeutic targets for treating MASLD and MASH: preclinical and clinical evidence.** Preclinical studies show that recombinant proteins like ORM2, ORM2-IgG Fc, IL-22, IL-22-IgG Fc, Ism1, Nrg4, GDF15, MANF, and FGF1, can potentially relieve hepatic steatosis. Moreover, ORM2, Nrg4, GDF15, MANF, FGF1, and SPARCL1 neutralizing antibody can attenuate MASH. In addition, Nrg4 can suppress the progression from MASH to hepatocellular carcinoma, and MANF can inhibit the development of hepatocellular carcinoma. Clinical trials reveal that the FGF19 analogue aldafermin can decrease liver fat content and fibrosis in MASH patients. The FGF21 analogue pegozafermin and efruxifermin can reduce liver fat, fibrosis, and injury in patients with severe hypertriglyceridemia, MASH, and liver fibrosis. Efruxifermin combined with GLP-1 receptor agonist therapy can reduce liver fat and fibrosis in MASH patients with T2DM. Additionally, the FGF19 analogue aldafermin and the FGF21 analogue efruxifermin can improve liver fibrosis and liver injury in patients with MASH-compensated cirrhosis. GLP-1 RA, GLP-1 receptor agonist; r, recombinant; SHTG, severe hypertriglyceridemia.

Secreted proteins play pivotal roles in regulating glucose and lipid metabolism through autocrine, paracrine and endocrine signaling. Pharmacotherapies based on secreted proteins, such as insulin analogues and GLP-1-like incretin mimetics, are integral to the treatment of diabetes or obesity. Over the past few decades, a growing number of secreted proteins with metabolic functions have been identified. These include hepatokines from the liver (Jensen-Cody and Potthoff, 2021), adipokines from adipose tissue (Jung and Choi, 2014), myokines from skeletal muscle (Chen et al., 2024c), cytokines from immune cells (Ouyang and O’Garra, 2019), and secreted proteins originating from the gut (Muskiet et al., 2017). These findings provide valuable opportunities for the development of new pharmaceutical therapies for metabolic diseases, including MASLD/MASH. While the molecular effects of many secreted proteins are currently under investigation, advancing our understanding of their clinical significance is equally important. In this review, we highlight the latest progress in understanding the roles of secreted proteins in hepatic TG metabolism, liver inflammation and fibrosis, and related metabolic disorders (Fig. 1). We also discuss their preclinical and clinical applications in treating MASLD/MASH (Fig. 2). These secreted proteins include the orosomucoid (ORM) family, secreted acidic cysteine rich glycoprotein (SPARC) family, neuregulin (Nrg) family, growth differentiation factor (GDF) family, interleukin (IL) family, fibroblast growth factor (FGF) family, bone morphogenic protein (BMP) family, as well as isthmin-1 (Ism1) and mesencephalic astrocyte-derived neurotrophic factor (MANF).

## Secreted proteins as therapeutic targets for treating MASLD/MASH

### Orosomucoids

Orosomucoids (ORMs) exist in three isoforms (ORM1, ORM2, and ORM3) in mice and two isoforms (ORM1 and ORM2) in humans. Among these, ORM1 constitutes approximately 75% of plasma ORMs and is mainly expressed in various peripheral tissues, including the liver (Luo et al., 2015). Traditionally, ORMs have been recognized as carriers of drugs and lipids; however, recent studies have revealed their important roles in metabolic regulation (Heo et al., 2024).

#### ORM1

ORM1 is primarily expressed in peripheral tissues, such as adipose tissue, liver, and skeletal muscle (Luo et al., 2015). ORM1-deficient mice exhibit exacerbated high-fat diet (HFD)-induced MASLD and methionine- and choline-deficient diet (MCD)-induced MASH, suggesting that endogenous ORM1 plays a protective role, although the underlying mechanism remains unclear. Interestingly, liver-specific overexpression of ORM1 fails to mitigate MASLD (Sun et al., 2025), indicating that the liver may not be the primary target for ORM1. Moreover, mice lacking ORM1 show increased fat mass, body weight, and hyperleptinemia. Conversely, exogenous administration of ORM, likely ORM1, significantly reduces food intake and body weight in obese mice by binding to the leptin receptor and activating the JAK2-STAT3 signaling in hypothalamic tissue (Sun et al., 2016). Additionally, ORM1 suppresses inflammation in adipocytes and macrophages, thereby maintaining energy homeostasis (Lee et al., 2010). Treatment with exogenous ORM inhibits adipogenesis and lipogenesis in adipocytes by suppressing multiple early-adipogenic transcription factors and interfering with C/EBPα and PPARγ activation, thereby preventing obesity (Lee et al., 2021). ORM1 is also considered as a myokine; in mice, ORM1 levels increase in skeletal muscle during fatigue, promoting muscle glycogen storage and enhancing muscle endurance via the C-C chemokine receptor type 5 (CCR5)-activated AMPK pathway (Qin et al., 2016). Overall, these findings suggest that ORM1 may improve MASLD/MASH through extrahepatic tissues such as adipose tissue and muscle.

Dysregulation of ORM1 has been linked to MASLD-related disorders. Elevated serum ORM levels have been observed in individuals, mice, and Ossabaw pigs with obesity (Alfadda et al., 2012; Bell et al., 2010; Lee et al., 2010; Sun et al., 2016, 2025). In humans, elevated ORM levels correlate with body mass index (BMI), body fat mass, and fasting plasma glucose levels (Gomes et al., 2003; Lee et al., 2010). Urinary ORM1 is positively correlated with hepatic fat content, homeostasis model assessment of insulin resistance (HOMA-IR), and liver injury parameters such as fibrosis and alanine aminotransferase (ALT) (Liu et al., 2022). A genome-wide association study suggests that ORM1 mediates the link between obesity and MASLD and impacts MASLD independently of obesity (Liu et al., 2023b). Collectively, these findings indicate that ORM1 plays a protective role against MASLD, although further research is required to fully elucidate its mechanisms.

#### ORM2

Orosomucoid 2 (ORM2) is an acute-phase secretory glycoprotein primarily produced by the liver, particularly in hepatocytes, in response to systemic injury (Luo et al., 2015). Like other acute-phase proteins, ORM2 expression is upregulated in response to many conditions, including inflammation, infection, tissue injury, and tumors (Heo et al., 2024; Hochepied et al., 2003; Huang and Ung, 2013; Irmak et al., 2009; Luo et al., 2015). Recent studies have identified ORM2 as a pivotal regulator of energy homeostasis, inflammation, immune response, and oxidative stress, primarily targeting hepatocytes and other extrahepatic tissues, including adipose tissue and the intestine (Han et al., 2010; Li et al., 2023, 2024; Wan et al., 2019; Zhou et al., 2022; Zhu et al., 2024). We recently found that both hepatic and circulating ORM2 levels were downregulated in mice and patients with MASLD/MASH (Zhou et al., 2022). *In vivo* studies demonstrated that ORM2 deficiency exacerbates HFD-induced hepatic steatosis and high-fat high-cholesterol (HFHC) diet-induced steatohepatitis in mice. Conversely, ORM2 overexpression mitigates hepatic steatosis and steatohepatitis and improves plasma lipid profiles. These beneficial effects are mainly attributed to the suppression of *de novo* lipogenesis as well as fatty acid uptake in hepatocytes, independent of body weight and food intake (Li et al., 2023; Zhang et al., 2024a; Zhou et al., 2022). Mechanistically, ORM2 binds to inositol 1,4,5-trisphosphate receptor type 2 (ITPR2) to activate AMP-activated protein kinase (AMPK) signaling, thereby inhibiting the sterol regulatory element binding protein 1c (SREBP-1c)-mediated lipogenic gene program (Zhou et al., 2022).

Hepatocyte-derived ORM2 also influences peripheral tissues, such as adipose tissue. In response to metabolic interventions, including intermittent fasting, bile acid treatment, and bariatric surgery, ORM2 expression is significantly increased. This induction promotes systemic metabolic effects by inducing adipose tissue browning and reducing inflammation in mice (Li et al., 2024; Zhu et al., 2024). Furthermore, although the exact mechanisms remain unclear, ORM2 deficiency has been associated with gut microbiome disturbances and the stimulation of intestinal inflammation in diet-induced obese mice (Li et al., 2024). Beyond peripheral tissues, ORM2 also targets the central nervous system, where it regulates neuronal mitochondrial biogenesis and promotes functional recovery after stroke (Jing et al., 2024).

Given its protective roles, ORM2 holds promise as a therapeutic target for MASLD/MASH and related metabolic diseases. Exogenous administration of recombinant ORM2 or long-acting ORM2-IgG Fc has been shown to attenuate hepatic steatosis, steatohepatitis, hyperlipidemia, and atherosclerosis in preclinical mouse models (Li et al., 2023; Zhou et al., 2022). Clinically, hepatic and plasma ORM2 levels are markedly reduced in obese murine models and patients with MASLD (Zhou et al., 2022). Therefore, multicenter clinical studies are necessary to solidify the connection between ORM2 expression and the development and progression of MASLD/MASH. Furthermore, future studies should assess the beneficial and potential adverse effects of ORM2 treatment in non-human primates before its use in clinic.

### Secreted acidic cysteine-rich glycoprotein family

The secreted acidic cysteine-rich glycoprotein (SPARC) family, a group of matricellular proteins, plays key roles in regulating cell proliferation and migration, thereby influencing processes, such as embryogenesis, tissue remodeling, and tumorigenesis (Klingler et al., 2020; Sullivan and Sage, 2004). This family includes molecules, such as SPARC, SPARCL-like 1 (SPARCL1), thrombospondin 1 and 2, and osteopontin, all of which have been implicated in the development of obesity, insulin resistance, and chronic liver diseases (Bai et al., 2020; Icer and Gezmen-Karadag, 2018; Kos and Wilding, 2010).

#### Secreted acidic cysteine-rich glycoprotein

Secreted acidic cysteine-rich glycoprotein (SPARC), also known as osteonectin or basement-membrane protein 40 (BM-40) (Ryu et al., 2022), is widely expressed in various tissues and organs, including adipose tissue, liver, and skeletal muscle (Kos and Wilding, 2010). As a multifunctional extracellular matrix (ECM) binding protein, SPARC is essential for cellular adhesion, ECM organization and remodeling, cellular growth and differentiation, wound repair, and fibrosis (Bradshaw and Sage, 2001; Rosset and Bradshaw, 2016). As a secreted protein, SPARC also plays a critical role in inter-tissue communication and metabolic regulation.

Induced SPARC expression has been observed in the fibrotic livers of both rodents and humans (Atorrasagasti et al., 2013; Blazejewski et al., 1997; Camino et al., 2008; Frizell et al., 1995; Mazzolini et al., 2018). The upregulation of SPARC in the liver is associated with elevated levels of liver injury and fibrosis markers, such as collagen Iα and transforming growth factor β (TGFβ), in both rodents and humans (Mazzolini et al., 2018). In murine fibrosis models, knockdown or knockout of SPARC markedly attenuates hepatic fibrosis, partly by suppressing the trans-differentiation of hepatic stellate cells (HSCs) into a myofibroblasts-like phenotype and inhibiting myofibroblasts activation (Atorrasagasti et al., 2013; Camino et al., 2008). Knockdown or knockout of SPARC also reduces inflammatory activity, partially by decreasing CD4^+^ T cells infiltration in the liver (Atorrasagasti et al., 2013; Camino et al., 2008). Furthermore, SPARC knockdown in activated HSCs significantly decreases platelet-derived growth factor-BB (PDGF-BB) or TGFβ-induced migration and reduces the expression of collagen and other ECM molecules (Atorrasagasti et al., 2011). These findings strongly suggest that SPARC upregulation is involved in fibrosis and positions it as a potential therapeutic target for liver fibrosis.

SPARC is also linked to hyperglycemia and insulin resistance. Elevated plasma SPARC levels have been detected in individuals with type 2 diabetes mellitus (T2DM) (Lee et al., 2013). *Sparc*-knockout mice exhibit abnormal insulin-regulated glucose metabolism, increased adipose tissue deposition, and impaired glucose homeostasis (Atorrasagasti et al., 2019). SPARC has also been implicated in alterations to pancreatic β-cell function. It promotes glucose-stimulated insulin secretion in pancreatic β cells (Harries et al., 2013; Hu et al., 2020). Correspondingly, the absence of SPARC leads to a decrease in GLUT2 expression in β cells, highlighting a defect in the glucose-sensing machinery (Atorrasagasti et al., 2019). Given its effects on lipid metabolism, fibrogenesis, inflammation, and insulin secretion, SPARC presents as an intriguing molecule to study in the context of MASLD and T2DM. Although little clinical studies have yet explored its therapeutic effects, its modulation could potentially impact metabolic diseases, including MASLD/MASH.

SPARC reduction also protects against obesity. In humans, a 2-year, 14% caloric restriction (CR) inhibits SPARC expression in adipose tissue (Ryu et al., 2022). Similarly, weight loss through CR and low-protein diet feeding in mice decreases SPARC expression, while HFD-induced obesity increases SPARC expression in adipose tissue (Ryu et al., 2023). Deletion of SPARC expression in adipocytes protects mice from HFD-induced adiposity, chronic inflammation, and metabolic disorders, and also reduces inflammation and prolongs health span during aging (Ryu et al., 2023). Mechanistically, SPARC activates the NLRP3 inflammasome and JNK signaling to promote inflammation via toll-like receptor 4 (TLR4) (Ryu et al., 2023). Additionally, SPARC dampens mitochondrial respiration in macrophages, and inhibition of glycolysis abolishes interferon-stimulated gene expression through transcription factors IRF3/7 (Ryu et al., 2022).

#### SPARC-like 1

SPARC-like 1 (SPARCL1) expression is highly enriched in white adipose tissue (WAT) and mature adipocytes (Gagliardi et al., 2017; Liu et al., 2021; Xiao et al., 2021) but is lower in the gastrointestinal tract, skeletal muscle, liver, kidney, spleen, and testis (Klingler et al., 2020). There may also be differences in SPARCL1 expression profiles between mice and humans. For example, SPARCL1 expression is relatively low in murine liver but moderate in human livers (Klingler et al., 2020). SPARCL1 has been shown to regulate numerous physiological processes, including cell adhesion, migration, proliferation, differentiation, and muscle development (Sakai et al., 2022; Sullivan and Sage, 2004; Wang et al., 2019c) and is considered a tumor suppressor in human cancers (Gagliardi et al., 2017).

Recent studies from our group and others have revealed a previously unrecognized role for SPARCL1 in MASLD/MASH (Liu et al., 2021; Meissburger et al., 2016; Xiao et al., 2021). SPARCL1 negatively impacts preadipocyte differentiation and suppresses lipid droplet accumulation by modulating peroxisome proliferator-activated receptor-gamma (PPARγ), CCAAT/enhancer-binding protein-alpha (C/EBPα), lipoprotein lipase (LPL), and insulin-like growth factor 1 (IGF1). This effect may lead to enhanced adipocyte hypertrophy in obesity (Meissburger et al., 2016; Xiao et al., 2021), suggesting that SPARCL1 controls adipogenesis in a paracrine/autocrine manner. As an adipokine, SPARCL1 exerts its metabolic regulatory functions through crosstalk with other organs such as the liver. Chronic recombinant SPARCL1 treatment induces hepatic steatosis, an inflammatory response, fibrosis, and liver injury in mice, to a greater extent than that observed in mice fed a 28-week high-fat high-cholesterol (HFHC) diet (Liu et al., 2021). Conversely, SPARCL1 deficiency, achieved through genetic manipulation or neutralizing antibodies, protects mice from long-term HFHC diet-induced hepatic inflammation and fibrosis (Liu et al., 2021). At the molecular level, SPARCL1 promotes the expression of C-C motif chemokine ligand 2 (CCL2) in hepatocytes, partly by binding to TLR4 and activating the NF-κB/p65 signaling pathway. Supporting this, another study reported that SPARCL1 is upregulated in liver-specific Apobec1 complementation factor transgenic mice, a model for steatosis, fibrosis, and hepatocellular carcinoma (Blanc et al., 2021).

SPARCL1 also regulates systemic glucose homeostasis. Recombinant SPARCL1 protein induces fasting hyperglycemia, hyperinsulinemia, and worsens insulin sensitivity in HFHC diet-fed mice. In contrast, SPARCL1-deficient mice are partially protected against HFHC diet-induced glucose dysregulation (Liu et al., 2021). *In vitro*, SPARCL1 treatment suppresses phosphorylation of AKT and GSK3β in hepatocytes, indicating an insulin-resistant state. SPARCL1 treatment also stimulates adipose tissue inflammation, which may exacerbate systemic insulin resistance (Huang et al., 2024). These findings highlight the metabolic hazards associated with SPARCL1, suggesting that blocking its action, such as through the use of neutralizing antibody, could serve as a potential therapy for treating MASLD/MASH.

Clinically, plasma SPARCL1 levels are significantly higher in patients with MASH than in normal individuals or patients with simple steatosis (Liu et al., 2021), in line with its role in driving steatosis-to-MASH progression. In the cohort study, plasma SPARCL1 levels are positively correlated with liver injury markers, such as ALT, aspartate aminotransferase (AST), and γ-glutamyl transferase (GGT) levels (Liu et al., 2021). Notably, the SPARCL1-ALT-AST model demonstrates improved accuracy compared to the ALT-AST model in identifying patients with MASH, suggesting its potential as a noninvasive diagnostic marker (Liu et al., 2021). However, prospective cohort studies are warranted to provide more evidence in humans.

### Neuregulins

Neuregulins (Nrgs) are a group of secreted proteins related to the epidermal growth factor (EGF) family, which act as ligands to activate tyrosine kinase receptors. The EGF receptors include EGFR (ErbB1), ErbB2, ErbB3, and ErbB4, while the Nrg family consists of Nrg1, Nrg2, Nrg3, and Nrg4 (Burden and Yarden, 1997). Nrgs are well known for their roles in development, nervous system regulation, and inter-organ communications (Cespedes et al., 2018).

#### Neuregulin 1

Nrg1 is ubiquitously expressed in endothelial and mesenchymal cells and plays a role in cell proliferation, survival, migration, and differentiation (Meyer et al., 1997). Nrg1 primarily binds to ErbB3 and ErbB4 to initiate downstream signaling. *In vitro* studies have shown that Nrg1 alleviates palmitic acid-induced lipid accumulation through ErbB3 phosphorylation and the PI3K-AKT signaling pathway (Meng et al., 2021). Similarly, Nrg1 mediates the beneficial effects of circular RNA ribonucleotide reductase subunit M2 (CircRRM2) in alleviating hepatic steatosis in mice (Wu et al., 2024). Nrg1 also impacts glucose homeostasis. In the liver, Nrg1 treatment activates AKT signaling (Caillaud et al., 2016), thereby reducing blood glucose levels, enhancing glucose tolerance (Ennequin et al., 2015), increasing insulin sensitivity (Guma et al., 2020), and suppressing food intake in obese or diabetic mice (Zhang et al., 2018). These metabolic benefits primarily result from inhibiting hepatic gluconeogenesis, restricting caloric intake via proopiomelanocortin (POMC) neurons (Zhang et al., 2018), improving mitochondrial respiration in skeletal muscle via complex 2 (Ennequin et al., 2017), and enhancing mitochondrial oxidation and insulin sensitivity in skeletal muscle (Canto et al., 2007). Furthermore, individuals with T2DM have substantially lower serum Nrg1 levels compared to controls (Al-Zuhairi et al., 2024; Eldin et al., 2023). Serum Nrg1 levels are negatively correlated with fasting blood glucose and hemoglobin A1c (HbA1c) levels, and insulin resistance (Eldin et al., 2023). These findings provide evidence for Nrg1’s potential as a therapeutic target for T2DM. However, due to the short half-life of native Nrg1 (Liu et al., 2006), a fusion protein (Nrg1-IgG Fc) with a longer half-life may be required for therapeutic applications.

#### Neuregulin 4

Neuregulin 4 (Nrg4) is an adipokine predominantly secreted by brown adipose tissue (BAT) and white adipose tissue (WAT) and primarily activates the ErbB4 receptor tyrosine kinase (Geissler et al., 2020; Harari et al., 1999; Villarroya et al., 2017; Wang et al., 2014a). Nrg4 exerts its effects primarily in adipose tissue and other metabolically active organs, such as the liver, hypothalamus, pancreas, skeletal muscle, and sympathetic neurons, in autocrine, paracrine, or endocrine manners (Gavalda-Navarro et al., 2022; Zhang et al., 2023b). Compelling evidence has established that Nrg4 plays a protective role in the development and progression of MASLD/MASH (Guo et al., 2017; Wang et al., 2014a; Zhang et al., 2022). Gain- and loss-of-function studies in mice have demonstrated that Nrg4 acts on hepatocytes to alleviate diet-induced hepatic steatosis by suppressing *de novo* lipogenesis through inhibiting LXR/SREBP1c signaling (Wang et al., 2014a). *In vitro* experiments using mouse primary hepatocytes further confirmed that Nrg4 dampens *de novo* lipogenesis via the ErbB4/STAT5/SREBP-1c axis (Li et al., 2021). Moreover, Nrg4 was shown to attenuate liver inflammation and injury through activation of ErbB4/AKT signaling and subsequent suppression of JNK-mediated hepatocyte death (Guo et al., 2017). In addition, a recent study found that Nrg4 can be induced by exercise to alleviate MASLD/MASH, which is also dependent on AKT signaling and suppression of cGAS-STING pathway-mediated inflammation and steatosis in hepatocytes (Chen et al., 2025). Nrg4 also acts as a checkpoint to suppress the progression from MASH to HCC by restraining the tumor-prone liver microenvironment (Zhang et al., 2022). These findings suggest that Nrg4 is involved in all stages of MASLD/MASH-HCC, highlighting the potential for Nrg4-based therapy in the treatment of these diseases.

In addition to its role in lipid metabolism, Nrg4 also regulates systemic glucose metabolism. Nrg4 deficiency impairs glucose and insulin tolerance and elevates blood glucose levels. Conversely, transgenic overexpression of Nrg4 can partly correct these abnormalities. In mice, Nrg4 exerts glucose-lowering effects partly through modulating gluconeogenesis in the liver and glucose utilization in skeletal muscle (Chen et al., 2017b; Wang et al., 2014a; Zhang et al., 2019). Nrg4/ErbB4 signaling also enhances insulin secretion in pancreatic β cells and glucose uptake in adipocytes (South et al., 2013; Zeng et al., 2018). Furthermore, recombinant Nrg4 protein has been shown to ameliorate age-associated glucose metabolic disorders. In the Nrg4-treated group, glucose tolerance and insulin resistance were improved, along with lower blood glucose and insulin levels compared to controls (Chen et al., 2024a).

The role of Nrg4 in obesity and whole-body energy homeostasis is gradually becoming clearer. Nrg4-null mice exhibit greater weight gain, increased fat mass, and decreased lean mass. In contrast, Nrg4 transgenic mice on an HFD have lower body weight and adiposity than controls, mediated by modulating beige fat thermogenesis (Chen et al., 2024b; Wang et al., 2014a). ErbB4-deletion mice also exhibit increased amounts of subcutaneous and visceral fat compared with wild-type mice (Zeng et al., 2018). Mechanistically, Nrg4 acts on the central nervous system to regulate body weight. Overexpression of Nrg4 in the paraventricular nucleus (PVN) of the brain protects against HFD-induced obesity, whereas ErbB4 knockdown in oxytocin neurons accelerates obesity (Zhang et al., 2023b).

Preclinical evidence shows that in obese mice, both Nrg4 expression in adipose tissue and hypothalamic ErbB4 phosphorylation are decreased (Wang et al., 2014a; Zhang et al., 2023b). Similarly, in individuals with high BMI, the mRNA expression levels of hepatic *ERBB4* and *NRG4* are reduced (Bograya et al., 2024), suggesting a protective role for Nrg4-ErbB4 signaling against obesity. Besides, circulating Nrg4 levels are lower in individuals with obesity (Cai et al., 2016; Guo et al., 2021), MASLD (Dai et al., 2015; Tutunchi et al., 2021; Wang et al., 2019a), and T2DM (Zhang et al., 2017), compared to healthy controls. There is also an inverse association between Nrg4 levels and metabolic syndrome indices, including BMI, waist circumference, fasting plasma glucose, TGs, HOMA-IR, and high-sensitivity C-reactive protein (Ziqubu et al., 2024). However, some studies have shown that Nrg4 levels are elevated in obese individuals (Martinez et al., 2022), which may reflect a compensatory response or resistance to its receptor (Wang et al., 2019b). Most of the current clinical evidence is cross-sectional or observational; therefore, large-sample, long-term prospective cohort studies are necessary to establish causality. Importantly, genetic studies have linked ErbB4 with T2DM and obesity (Boger and Sedor, 2012; Locke et al., 2015; Maeda et al., 2013; Sandholm et al., 2012). In addition, we recently identified two human Nrg4 variants, R44H and E47Q, in severely obese individuals. Nrg4 E47Q can activate ErbB4 phosphorylation and inhibit *de novo* lipogenesis via the ErbB4/STAT5/SREBP-1c pathway. In contrast, Nrg4 R44H has lost this function, thereby promoting lipogenesis and metabolic disorders (Li et al., 2021).

### Growth differentiation factor family

The growth differentiation factor (GDF) family is part of the TGFβ superfamily and encompasses several members, such as GDF15 and GDF10 (Herpin et al., 2004). These proteins interact with distinct type I and type II serine/threonine kinase receptors (Rochette et al., 2020) and are involved in a variety of pathophysiological processes.

#### GDF15

Growth differentiation factor 15 (GDF15), a member of the TGFβ superfamily (Hsiao et al., 2000), is expressed in most body cell types and is induced under stress conditions (Sigvardsen et al., 2024). GDF15 functions via the orphan GFRα family member GFRAL and signals through the Ret coreceptor (Emmerson et al., 2017; Mullican et al., 2017).

Initially, GDF15 was shown to regulate appetite control and body weight through central actions (Tsai et al., 2014). Activation of the GDF15/GFRAL axis induces anorexia and weight loss (Emmerson et al., 2017; Hsu et al., 2017; Mullican et al., 2017; Yang et al., 2017). Transgenic *Gdf15*-overexpressing mice exhibit reduced food intake, a lean phenotype, and resistance to obesity, metabolic inflammation, and glucose intolerance (Chrysovergis et al., 2014; Macia et al., 2012; Wang et al., 2014b, 2014c). Similarly, ablation of *Gdf15* or *Gfral* leads to increased fat depots and body weight in HFD-induced obese mice (Hsu et al., 2017; Tran et al., 2018). Recombinant GDF15 limits food intake and body weight in mice, but these effects are absent in *Gfral*-deficient mice (Emmerson et al., 2017; Hsu et al., 2017; Yang et al., 2017). Besides, recent discoveries found that this signaling is also required for ketogenic diet (KD)-induced weight loss (Breit et al., 2023; Lu et al., 2023).

Beyond its central actions, GDF15 also directly targets peripheral tissues such as the liver and skeletal muscle, enhancing oxidative metabolism and lipid mobilization (Chung et al., 2017), increasing thermogenic and lipolytic gene expression in adipose tissue (Chrysovergis et al., 2014), and ameliorating proinflammatory cytokine-induced pancreatic β cell apoptosis (Nakayasu et al., 2020). GDF15 also enhances insulin action in the liver and adipose tissue via a β adrenergic receptor-mediated mechanism (Sjoberg et al., 2023). In patients with MASLD/MASH, GDF15 levels are elevated and increase with disease progression, suggesting that GDF15 may serve as a novel biomarker (Koo et al., 2018). The endogenous induction of GDF15 may represent an adaptive and compensatory response to mitigate MASH progression (Breit et al., 2021; Kim et al., 2018). In diet-induced MASH mice, GDF15 ablation exacerbates hepatic steatosis, inflammation, fibrosis, and liver injury, while transgenic GDF15 expression alleviates these phenotypes and metabolic deterioration (Kim et al., 2018; Wang et al., 2022b). Consistently, recombinant GDF15 treatment significantly reduces hepatic steatosis in mice (Chung et al., 2017). Besides, upon KD feeding, *Gdf15* mRNA levels increase in hepatocytes, elevating circulating GDF15 levels, with all MASLD-related changes occurring without altering food intake. Additionally, *Gdf15* or *Gfral* ablation abolishes the beneficial effects of the KD on reducing hepatic lipids and plasma ALT/AST levels in mice (Lu et al., 2023). The anti-lipogenic, anti-fibrogenic, and anti-inflammatory effects of GDF15 are attributed to enhanced hepatic fatty acid β-oxidation (Kim et al., 2018; Li et al., 2018), suppression of fibrosis-related gene expression in HSCs (Kim et al., 2018; Wang et al., 2022b), inhibition of fatty acid uptake and synthesis, and dampening of AIM2 inflammasome activation (Wang et al., 2022b). These findings suggest that GDF15 directly affects the liver to mitigate MASLD/MASH.

GDF15 holds promise as a therapeutic target for metabolic disorders, with encouraging preclinical results. However, in the Phase 1 study of LY3463251 (a long-acting GDF15 analog), there was only a modest impact on weight loss in obese individuals, although it significantly reduced food intake and appetite (Benichou et al., 2023). Similarly, in the Phase 2 study of MBL949 (a recombinant human GDF15 dimer with an extended half-life), biweekly dosing for 14 weeks resulted in minimal weight loss. Both clinical studies suggest that the robust weight loss effects observed in nonclinical models did not translate into significant weight loss efficacy in humans (Smith et al., 2024). Thus, more clinical trials are required to fully assess its therapeutic potential in humans. Additionally, combining GDF15 with GLP-1 treatment may represent a promising strategy for weight loss, as synergistic effects of these two proteins have been observed in mice and non-human primates (Zhang et al., 2023a). However, it remains to be determined if this combination is effective in humans.

#### GDF10

Growth differentiation factor 10 (GDF10), also known as BMP-3b, is an atypical member of the TGFβ superfamily (Matsumoto et al., 2012). In diet-induced hepatic steatosis, circulating GDF10 levels are elevated (Platko et al., 2019). GDF10 prevents excessive lipid accumulation in hepatocytes by reducing nuclear PPARγ activity. When GDF10 is ablated in mice, liver TG deposition increases, and an obese phenotype develops even on a normal diet (Platko et al., 2019). In response to an HFD, GDF10-ablated mice exhibit increased steatosis, ER stress, fibrosis, and liver injury compared to controls (Platko et al., 2019). These findings highlight GDF10’s hepatoprotective role, as it inhibits *de novo* lipogenesis and protects against liver injury. GDF10 is also required for maintaining glucose metabolism homeostasis during aging. GDF10 knockout mice develop glucose intolerance and insulin resistance, along with elevated plasma cholesterol and TG levels (Marti-Pamies et al., 2020).

#### GDF3

In the liver, we found that GDF3 was predominantly expressed in Kupffer cells and macrophages (Xiang et al., 2022). Hepatic GDF3 expression and plasma GDF3 concentrations were specifically upregulated in MASH mice, while they remained unchanged in mice with simple steatosis (Xiang et al., 2022). Consistently, plasma GDF3 levels are significantly elevated and correlated with hepatic pathological features in patients with MASH, offering high diagnostic accuracy for MASH with good sensitivity and specificity (Xiang et al., 2022). In addition, GDF3 expression has been associated with obesity and diabetes (Bu et al., 2018; Izumi, 2023), and its ectopic expression can induce insulin resistance in lean and healthy mice (Hall et al., 2020). Further research is needed to investigate the mechanism by which GDF3 contributes to MASH pathogenesis.

### Interleukins

Interleukins (IL) are produced by various immune cells in both the innate and adaptive immune systems. They are crucial messenger molecules that regulate biological functions of target cells through autocrine or paracrine mechanisms (Ouyang and O’Garra, 2019).

#### Interleukin-22

Interleukin-22 (IL-22), a member of the IL-10 cytokine family, is primarily produced by cells of the adaptive immune system and innate lymphoid cells (Ouyang et al., 2011). Its functional receptor, IL-22RA1, is expressed in various organs and tissues, including the liver, pancreas, kidney, intestine, adipose tissue, and skin (Dudakov et al., 2015). IL-22RA1 pairs with IL-10RB to mediate IL-22 signaling, primarily through signal transducer and activator of transcription (STAT) pathway (Dudakov et al., 2015). Initially recognized for its roles in tissue protection, immune regulation, and tissue regeneration (Dudakov et al., 2015), emerging evidence now highlights IL-22’s versatile functions in metabolic regulation and its potential for improving MASLD/MASH.

Through multiple mouse models, IL-22 has been shown to alleviate hepatic steatosis and injury by inhibiting lipogenesis (Gaudino et al., 2024; Huang et al., 2025; Sajiir et al., 2024a; Wang et al., 2014d; Yang et al., 2010; Zhang et al., 2024b). In contrast, hepatocyte-specific IL-22RA1 knockout mice exhibited diet-induced hepatic steatosis and liver injury, primarily driven by elevated lipogenesis and hepatic oxysterol accumulation (Huang et al., 2025). Mechanistically, we found that hepatic IL-22RA1 deficiency impairs STAT3 signaling, which leads to the upregulation of activating transcription factor 3 (ATF3). Subsequently, ATF3 inhibits oxysterol 7 alpha-hydroxylase (CYP7B1) expression, causing oxysterol accumulation to activate LXRα, resulting in the transactivation of SREBP-1c and lipogenic genes (Huang et al., 2025). IL-22 also mitigates hepatic fibrosis by targeting HSCs (Hwang et al., 2020; Kong et al., 2012; Zhang et al., 2024b), Kupffer cells (KCs), and monocyte-derived macrophages (Zhang et al., 2024b). It reduces liver inflammation and fibrosis induced by carbon tetrachloride (CCl4) (Kong et al., 2012), neutrophil-driven inflammation (Hwang et al., 2020), and diet-induced liver injury (Hwang et al., 2020; Zhang et al., 2024b), and aids in the resolution of liver fibrosis during recovery (Kong et al., 2012). In agreement, hepatocyte-specific IL-22RA1 deficiency exacerbates diet-induced inflammation and fibrosis in mice (Huang et al., 2025). IL-22 attenuates fibrosis through multiple ways, including blocking hepatic oxidative stress and related stress kinases (Hwang et al., 2020), inducing HSC senescence via p53 and p21-dependent pathways (Kong et al., 2012), reducing HSC activation and expansion, and decreasing M1-type Kupffer cells and monocyte-derived macrophages (Zhang et al., 2024b). In addition, IL-22 mitigates the inflammatory functions of hepatocyte-derived, mitochondrial DNA-enriched extracellular vesicles, thereby suppressing liver inflammation in MASH (Hwang et al., 2020). IL-22 also helps maintain intestinal homeostasis to alleviate MASLD. Obesogenic diets suppress IL-22 expression in small intestine innate lymphoid cells, inhibiting STAT3 signaling in intestinal epithelial cells (IECs) and expanding the absorptive enterocyte compartment. In IECs, exogenous IL-22 binds to its receptor, activates STAT3 and inhibits WNT/β-catenin signaling, thus exerting therapeutic effects (Zhang et al., 2024b). Furthermore, IL-22RA1 signaling deficiency in IECs promotes hepatocyte ballooning and lipid droplet deposition in a microbiota-dependent manner, suggesting that IECs might be required for the anti-steatotic roles of IL-22 (Gaudino et al., 2024). Notably, a recent study showed that the effects of IL-22 appear to be sex-dependent. In MASLD, females exhibit higher hepatic IL-22 expression, and the absence of IL-22RA1 signaling worsens hepatic pathology more severely in females (Abdelnabi et al., 2022).

In extrahepatic tissues, IL-22 enhances insulin secretion by reducing β cell oxidative and ER stress (Hasnain et al., 2014; Sajiir et al., 2024b), improving mitochondrial function, and maintaining β cell identity (Yu et al., 2024). IL-22 signaling suppresses inflammation and promotes high-quality insulin secretion, thereby restoring glucose homeostasis in diabetic obese models (Hasnain et al., 2014). Consistently, blocking IL-22RA1 in healthy mouse islets leads to increased ER stress and reduced glucose-stimulated insulin secretion (GSIS). In mice with pancreatic β cell IL-22RA1 deficiency, hyperglycemia, defective insulin secretion, impaired glucose tolerance, and decreased insulin quality are observed (Sajiir et al., 2024b; Yu et al., 2024). Similar to its effects on lipid metabolism, the effects of IL-22 on glucose regulation seem to be sex-dependent, although some conflicting findings exist (Sajiir et al., 2024b; Yu et al., 2024). Beyond insulin secretion, IL-22 also improves insulin sensitivity in muscle and adipose tissues upon insulin challenge (Wang et al., 2014d). Likewise, IL-22 treatment enhances glucose tolerance and insulin sensitivity in both diet- and genetically induced obese mice (Huang et al., 2025; Wang et al., 2014d). Animal models with global, hepatocyte-, or IEC-specific IL-22RA1 knockout show worsened obesity-associated insulin resistance and/or glucose intolerance (Gaudino et al., 2024; Huang et al., 2025; Wang et al., 2014d; Zhang et al., 2024b). Nevertheless, WAT-specific IL-22RA1 deficiency does not influence metabolic disorders (Gaudino et al., 2024; Wang et al., 2014d), suggesting that IL-22 exerts its metabolic effects in a context-dependent manner.

Given its pleiotropic functions, including anti-steatotic, anti-inflammatory, and anti-fibrotic effects within the liver, IL-22 is a promising candidate for treating MASLD/MASH (Huang et al., 2025). Multiple preclinical studies show that recombinant IL-22 or IL-22Fc can reduce hepatic steatosis, fibrosis, and inflammation, and attenuate weight gain in diet-induced obese mice (Hwang et al., 2020; Wang et al., 2014d; Yang et al., 2010; Zhang et al., 2024b). Furthermore, recombinant IL-22 can reverse glucose intolerance, hyperglycemia, and insulin resistance in obese mice (Hasnain et al., 2014; Wang et al., 2014d). Liver-targeted IL-22 gene delivery via nanoparticles also alleviates HFD-induced hepatic steatosis, hyperglycemia, and insulin resistance (Zai et al., 2019). IL-22-bispecific biologics targeting the liver and pancreas can reduce hepatic steatosis, inflammation, and fibrosis, and restore glycemic control in MASH mice (Sajiir et al., 2024a). Clinical studies support these findings, as newly diagnosed T2DM patients show lower circulating IL-22 levels (Asadikaram et al., 2018), suggesting that lower IL-22 levels may be associated with higher T2DM risk. However, patients with established metabolic syndrome, cardiometabolic disorders, and/or T2DM exhibit elevated circulating IL-22 levels, potentially as a compensatory response (Asadikaram et al., 2018; Gong et al., 2016; Gu et al., 2022).

F-652, a recombinant fusion protein of human IL-22 and IgG2 Fc, has undergone clinical trials, demonstrating safety and minimal adverse effects in Phase I trials (Tang et al., 2019). Phase IIa trials showed safety and efficacy in severe alcoholic hepatitis, reducing inflammation and injury markers, and promoting hepatic regeneration (Arab et al., 2020). However, gastrointestinal and dermatological side effects were noted, likely due to IL-22’s proliferative effects on epithelial tissues (Arshad et al., 2020; Moniruzzaman et al., 2019). The understanding of IL-22/IL-22RA1 signaling in metabolic health is advancing, highlighting its therapeutic potential. IL-22 exhibits both protective and pathogenic effects (Dudakov et al., 2015) and regulates tissue regeneration (Dudakov et al., 2015; Lindemans et al., 2015). Paradoxically, supraphysiological IL-22 levels have been shown to increase susceptibility to tumorigenesis (Jiang et al., 2011), while another study reports no spontaneous development of liver tumors (Park et al., 2011). Therefore, targeted delivery, structure-based design, precise modulation strategies, and combination therapies could help improve efficacy and minimize off-target effects, thereby unlocking IL-22’s therapeutic potential for treating MASLD/MASH.

#### Interleukin-18

Interleukin-18 (IL-18) is produced by various cell types, including Kupffer cells, hematopoietic cells, and non-hematopoietic cells such as intestinal epithelial cells, keratinocytes, and endothelial cells (Arend et al., 2008; Kaplanski, 2018). IL-18 binds to its specific receptor, which comprises the IL-18Rα and IL-18Rβ chains, triggering intracellular signaling pathways similar to those of IL-1 and activating NF-κB and inflammatory processes (Robinson et al., 1997). In the liver, IL-18 is primarily produced by macrophages (Belkaya et al., 2019; Lebel-Binay et al., 2000) and mediates the liver’s defense against bacterial, parasitic, viral infections, and drug-induced injuries (Imaeda et al., 2009; Maltez et al., 2015; Serti et al., 2014). Recent studies have implicated IL-18 signaling in hepatic steatosis and fibrosis. IL-18 is induced upon NLRP1 activation and decreased upon NLRP1 loss (Murphy et al., 2016), and its production could prevent hepatic steatosis (Murphy et al., 2016). Consequently, the loss or gain of NLRP1 function recapitulates obesity-related phenotypes in mice lacking or overexpressing IL-18 (Murphy et al., 2016). Mice with constitutively activated NLRP1, having elevated IL-18 levels, do not develop liver lipid accumulation, and IL-18 depletion reverses this protective effect (Murphy et al., 2016; Netea and Joosten, 2016). Exogenous IL-18 administration has been shown to counteract steatohepatitis in mice (Murphy et al., 2016), whereas IL-18-deficient mice exhibit hepatic steatosis and steatohepatitis (Lana et al., 2016; Netea et al., 2006; Yamanishi et al., 2016). Furthermore, elevating IL-18 levels by disrupting macrophage phosphatase SHP2 protects mice from HFD-induced hepatic steatosis (Liu et al., 2020b). The inhibitory effects of IL-18 on liver lipid storage and fibrosis may arise from its direct regulation of lipid homeostasis, including anti-hypercholesterolemia and anti-hypertriglyceridemia effects (Murphy et al., 2016), as well as from indirect effects, such as anti-hyperphagia and resulting anti-obesity effects (Netea et al., 2006) and modulation of gut microbiota composition (Henao-Mejia et al., 2012).

Conversely, other studies suggest that IL-18 can enhance hepatic fibrosis and injury. Recombinant IL-18 protein accelerates the trans-differentiation of primary murine HSCs into myofibroblasts, promoting ECM deposition and contributing to liver fibrosis (Knorr et al., 2023). *In vivo*, IL-18 receptor-deficient mice show reduced liver fibrosis in an HSC-specific NLRP3 overactivation-induced fibrosis model (Knorr et al., 2023). Similarly, IL-18R-deficient mice, but not IL-1R-deficient mice, are protected from early liver damage (Hohenester et al., 2020). These findings highlight the complex role of IL-18 in the liver, demonstrating a balance between its protective effects and potential fibrogenic risks. IL-18 also plays a role in glucose homeostasis. IL-18-deficient mice exhibit hyperinsulinemia, consistent with insulin resistance related to hyperglycemia, primarily due to enhanced liver gluconeogenesis and defective STAT3 phosphorylation (Netea et al., 2006). Additionally, IL-18 mediates the positive effects of the Nlrp1b inflammasome on glucose tolerance and insulin sensitivity (Salazar-Leon et al., 2019).

Clinically, circulating IL-18 levels are linked to increased liver injury markers (AST, ALT) and portal fibrosis (Lopez-Bermejo et al., 2005; Mehta et al., 2014). Patients with MASLD and chronic liver disease have higher plasma IL-18 levels (Li et al., 2010; Ludwiczek et al., 2002). IL-18 polymorphisms may contribute to susceptibility to certain types of chronic liver diseases (Zhang et al., 2020). Many clinical studies report upregulated IL-18 circulating levels in patients with T2DM (Moriwaki et al., 2003; Zaharieva et al., 2018; Zhuang et al., 2019). Prediabetic patients have higher IL-18 levels than obese normoglycemic controls (Gateva et al., 2020). Additionally, circulating IL-18 levels are positively correlated with the HOMA-IR index (Fischer et al., 2005). Conversely, a decrease in IL-18 has been identified as an independent factor for the improvement of β cell function in T2DM (Kim et al., 2007). An IL-18 gene polymorphism associated with higher circulating IL-18 levels is linked to impaired insulin sensitivity (Presta et al., 2009). High blood IL-18 levels in T2DM may indicate IL-18 resistance, akin to insulin resistance.

### Fibroblast growth factors

The fibroblast growth factor (FGF) family consists of 22 structurally related proteins that play diverse roles in cell proliferation, differentiation, embryonic development, wound repair, and angiogenesis (Beenken and Mohammadi, 2009). Among its members, two endocrine FGFs (FGF21 and FGF19) and the canonical FGF1 are important regulators of glucose and lipid metabolism, as well as energy homeostasis.

#### Fibroblast growth factor 21

Fibroblast growth factor 21 (FGF21) was initially identified as a prototypical hepatokine released primarily by the liver. Subsequent studies showed that circulating FGF21 levels are derived from both the liver and adipose tissue in rodents (Markan et al., 2014) and humans (Hansen et al., 2015). A variety of nutritional factors (e.g. fasting, high-carbohydrate, low-protein/amino acid levels) (Flippo and Potthoff, 2021; Potthoff, 2017) and stress signals (e.g. cold exposure, alcohol consumption) (BonDurant and Potthoff, 2018; Fisher and Maratos-Flier, 2016; Jensen-Cody and Potthoff, 2021; Soberg et al., 2018) strongly induce FGF21 expression. FGF21 elicits its biological effects by binding to and activating a receptor complex consisting of the co-receptor β-Klotho (KLB) and fibroblast growth factor receptor 1c (FGFR1c). KLB expression is primarily restricted to specific metabolic tissues, such as the liver, pancreas, and adipose tissue, determining the specificity of FGF21 signaling (Kurosu et al., 2007; Suzuki et al., 2008). FGF21 influences glucose and lipid metabolism and energy homeostasis by targeting diverse tissues, including the liver, pancreas, adipocytes, skeletal muscle, and brain (Bookout et al., 2013; Jensen-Cody et al., 2020; Liang et al., 2014). The most prominent and consistent metabolic effect of FGF21 across species is its impact on lipid metabolism (Markan and Potthoff, 2016). The liver is both the primary source and target of FGF21. In obese rodents, FGF21 exerts potent TG-lowering effects in the liver and plasma (Ahuja et al., 2023; Badman et al., 2007; Byun et al., 2020; Fisher et al., 2014; [Bibr CIT0085][Bibr CIT0164]; Xu et al., 2009a; Zarei et al., 2018). It achieves this mainly by increasing hepatic fatty acid oxidation, inhibiting lipogenesis, promoting the browning of WAT, and enhancing whole-body energy expenditure. Additionally, FGF21 reduces hepatic ER stress and oxidative stress (Fisher et al., 2014; Kim et al., 2015; Ye et al., 2014; Zarei et al., 2018), restricts liver inflammation and injury (Feingold et al., 2012; Fisher et al., 2014), and mitigates fibrosis in mice (Fisher et al., 2014; Ji et al., 2024; Liu et al., 2023a). Moreover, treatment with LY2405319, an engineered FGF21 analogue, significantly decreases MASH scores, serum liver fibrosis markers, injury markers, and pro-inflammatory markers in mice with MASH (Lee et al., 2016). These anti-lipogenic, anti-inflammatory, and anti-fibrotic effects are attributed to targeting both hepatocytes and HSCs in the liver (Harrison et al., 2024b). In hepatocytes, recent findings have identified serine and threonine phosphatase PPP6C as a direct target of FGF21. Mechanistically, PPP6C is sufficient to bind with the coreceptor βKlotho upon FGF21 treatment, directly dephosphorylating tuberous sclerosis complex 2 (TSC2) at Ser939 and Thr1462, thereby inhibiting mTORC1 activation (Liu et al., 2025). Thus, these results clearly demonstrate a fundamental and critical mechanism FGF21 action in hepatocytes to ameliorate MASLD/MASH. Furthermore, FGF21 also regulates lipid metabolism in adipose tissues by lowering plasma TGs through facilitating lipoprotein catabolism in WAT and BAT (Schlein et al., 2016).

Beyond lipid regulation, FGF21 is a potent acute insulin-sensitizer and a potential modulator of glucose metabolism. Mice lacking FGF21 exhibit elevated sugar intake, while FGF21 overexpression inhibits sugar intake (von Holstein-Rathlou et al., 2016). A single FGF21 injection can reduce plasma glucose by more than 50% in obese animal models (BonDurant et al., 2017; Xu et al., 2009b). Chronic FGF21 administration improves insulin sensitivity and glycemic control in obese animal models (Berglund et al., 2009; Coskun et al., 2008; Kharitonenkov et al., 2005; Xu et al., 2009a). Plasma glucose reduction primarily occurs through peripheral glucose disposal (BonDurant et al., 2017; Feingold et al., 2012; Xu et al., 2009b), particularly in adipose tissues (BonDurant et al., 2017; Lan et al., 2017). The anti-hyperglycemia effects of FGF21 have been replicated in diabetic monkey models (Adams et al., 2013; Kharitonenkov et al., 2007; Stanislaus et al., 2017; Talukdar et al., 2016), without inducing mitogenesis or hypoglycemia in rodents or monkeys. FGF21 also improves insulin sensitivity in various tissues, including the liver, adipose tissues, and skeletal muscle, thereby enhancing glucose homeostasis (Geng et al., 2020).

Prolonged administration of FGF21 or its analogues results in significant weight loss in rodents and non-human primates (BonDurant and Potthoff, 2018; Coskun et al., 2008; Kharitonenkov et al., 2005, 2007; Talukdar et al., 2016; Xu et al., 2009a). PF-05231023, a long-acting FGF21 analogue, leads to notable weight loss in obese monkeys and humans (Talukdar et al., 2016). The weight-loss effects of FGF21 or its analogue are mediated through direct actions on the central nervous system to facilitate energy expenditure (Ameka et al., 2019; Flippo et al., 2020; Hill et al., 2019; Lan et al., 2017; Owen et al., 2014), rather than through direct actions on adipose tissues (BonDurant et al., 2017; Chen et al., 2017a; Lan et al., 2017), although the underlying mechanism remains incompletely understood.

FGF21 analogues are under investigation for metabolic diseases, such as obesity, dyslipidemia, T2DM, and MASLD/MASH (Gaich et al., 2013; Harrison et al., 2021b, 2023a, 2023b; Loomba et al., 2023b; Rader et al., 2022; Sanyal et al., 2019). While their efficacy in glycemic control and weight loss is mild and inconsistent (Chui et al., 2024), benefits in dyslipidemia, hepatic steatosis, and MASH resolution have been observed. Efruxifermin (Harrison et al., 2025) and pegozafermin (Bhatt et al., 2023) met Phase IIb endpoints in patients with MASH. Efruxifermin reduces liver fat fraction (Harrison et al., 2021b), improves liver histology (Harrison et al., 2023a) and fibrosis (Harrison et al., 2023b), enhances lipid profiles and glycemic control, and may aid in weight loss (Harrison et al., 2023a). It also improves fibrosis and liver injury biomarkers in patients with compensated MASH cirrhosis; 57% of treated patients showed fibrosis improvement or resolution of MASH, compared to 0% in the placebo group (Harrison et al., 2023b). Pegozafermin, a long-acting glycol-pegylated recombinant FGF21 analogue, effectively reduces liver fat and fibrosis in patients with severe hypertriglyceridemia, MASH, and liver fibrosis. (Bhatt et al., 2023; Loomba et al., 2023a, 2023b). In addition to monotherapy, combination therapy shows strong strength. In a Phase II clinical trial, the combination of efruxifermin and GLP-1 receptor agonist (GLP-1RA) (Harrison et al., 2025) achieved strong hepatic fat reduction in patients with MASH, fibrosis, and T2DM. Efruxifermin improves noninvasive markers of liver injury, fibrosis, glucose, and lipid metabolism, maintains GLP-1RA-mediated weight loss, and is safe and well-tolerated. During clinical studies, no significant changes in bone density (Loomba et al., 2023b, 2024) or increased fracture incidence were reported (Abdelmalek et al., 2024). Phase III programs for efruxifermin and pegozafermin are underway. Thus, FGF21 analogues show promise for MASH treatment due to their pleiotropic effects in hepatocytes, anti-fibrotic properties, and systemic benefits.

#### FGF19

Fibroblast growth factor 19 (FGF19, known as FGF15 in rodents), primarily expressed in ileal enterocytes (Somm and Jornayvaz, 2018), is released into the enterohepatic circulation postprandially in response to bile acids via activation of the farnesoid X receptor (FXR) (Walters, 2014). FGF19’s involvement in hepatic fat metabolism was first recognized in 2002. Transgenic mice overexpressing FGF19 show reduced hepatic fat accumulation, fat mass, and body weight (Tomlinson et al., 2002). Similarly, FGF15-deficient mice exhibit exacerbated hepatic steatosis, whereas FGF19 treatment reverses this condition (Alvarez-Sola et al., 2017). Recombinant FGF19 or its analogue can resolve steatohepatitis and fibrosis in mice (Zhou et al., 2017). Mechanistically, FGF19 regulates hepatic fat metabolism via multiple pathways. It represses hepatic *de novo* lipogenesis (Bhatnagar et al., 2009; Fu et al., 2004; Tomlinson et al., 2002), increases the metabolic rate (Fu et al., 2004), inhibits ER stress (Alvarez-Sola et al., 2017), and reduces lipotoxicity (Alvarez-Sola et al., 2017; Zhou et al., 2017). These processes primarily involve the activation of signal transducer and activator of transcription 3 (STAT3) and the inhibition of peroxisome proliferator-activated receptor coactivator-1β (PGC-1β) and sterol regulatory element-binding protein 1c (SREBP-1c) (Sciarrillo et al., 2021). Furthermore, FGF19 may influence fat metabolism in tissues other than the liver (Potthoff et al., 2012). In addition to regulating lipid homeostasis, FGF15/19 also plays a role in carbohydrate metabolism. *Fgf15*-knockout mice exhibit impaired serum glucose regulation, reduced hepatic glycogen content, and glucose intolerance, while FGF19 administration reverses these metabolic impairments (Kir et al., 2011). Mechanistically, FGF15/19 promotes hepatic glycogen synthesis via extracellular-signal-regulated kinase (ERK) activation and glycogen synthase kinase-3 (GSK3) phosphorylation/inactivation, independent of insulin (Kir et al., 2011). FGF19 also inhibits gluconeogenesis by dephosphorylating and inactivating cyclic adenosine monophosphate (cAMP) regulatory element-binding protein (CREB) (Kir et al., 2011; Potthoff et al., 2011).

In healthy individuals, circulating FGF19 levels exhibit diurnal fluctuations closely associated with postprandial serum bile acids (Lundasen et al., 2006). Reduced fasting plasma levels of FGF19 have been observed in subjects with obesity, T2DM, and MASLD (Alisi et al., 2013; Eren et al., 2012; Mraz et al., 2011). Conversely, hepatic KLB and FGFR4 expression are increased in patients with MASLD/MASH, suggesting potential disruption in the FGF19/KLB/FGFR4 signaling pathway (DePaoli et al., 2019). Aldafermin (also known as M70 and NGM282), an engineered FGF19 analogue, is undergoing extensive clinical trials for treating metabolic liver diseases (Zhou et al., 2014). Aldafermin treatment leads to rapid and sustained reductions in liver fat content, liver injury and fibrosis markers, and improved liver histology in patients with MASH (Harrison et al., 2018, 2020, 2021a). However, a Phase 2b study (ALPINE 2/3, NCT03912532) involving patients with fibrosis stage 2 or 3 reported that aldafermin did not reach pre-specified significance, although significant improvements in steatosis, inflammation, liver injury, and fibrosis were achieved (Harrison et al., 2022). In a recently reported Phase 2b trial (NCT04210245) involving patients with compensated MASH cirrhosis, aldafermin treatment led to a significant reduction in enhanced liver fibrosis (Rinella et al., 2024). Additionally, aldafermin fails to reduce hyperglycemia in patients with T2DM (DePaoli et al., 2019). Thus, more clinical trials are needed to further validate its therapeutic effects.

#### FGF1

FGF1 is an autocrine and paracrine regulator that binds to heparan sulfate proteoglycans, preventing its secretion into circulation (Beenken and Mohammadi, 2009). Although FGF1 is involved in processes, such as embryonic development, wound healing, angiogenesis, and neurogenesis, global *Fgf1* knockout mice show no significant deficiencies in these processes (Miller et al., 2000). Recently, FGF1 has been found to exert an unexpected metabolic role in regulating lipid metabolism and glucose homeostasis in obesity, MASLD, and diabetes (Jonker et al., 2012; Suh et al., 2014). Chronic FGF1 treatment alleviates hepatic steatosis, inflammation, apoptosis, and oxidative stress in *ob*/*ob*, *db*/*db*, and high-fat/high-sucrose diet-induced MASLD model mice (Lin et al., 2021; Suh et al., 2014; Xu et al., 2020). FGF1 also reverses high-fat/high-sucrose diet-induced steatohepatitis and fibrosis in apolipoprotein E knockout mice and choline-deficient diet-fed mice through FGFR4-mediated AMP-activated kinase α (AMPKα) signaling (Lin et al., 2021; Liu et al., 2016), highlighting FGF1’s importance in maintaining hepatic lipid homeostasis.

FGF1 also plays a unique role in glucose homeostasis. A single-dose parenteral delivery of recombinant FGF1 normalizes blood glucose in obese mice without causing hypoglycemia (Suh et al., 2014). Chronic recombinant FGF1 treatment sustains glucose-lowering effects by promoting insulin-dependent glucose uptake in skeletal muscle and suppressing hepatic glucose production (HGP), thereby achieving insulin sensitization (Suh et al., 2014). The glucose-lowering and insulin-sensitizing effects of recombinant FGF1 are partially mediated by FGFR1 in adipose tissue (Suh et al., 2014). Mechanistically, FGF1 inhibits lipolysis in adipose tissue and suppresses HGP (Sancar et al., 2022). FGF1 also exerts central actions, as it crosses the blood–brain barrier (Lou et al., 2012) and is involved in feeding-suppression regulation (Suzuki et al., 2001). A single intracerebroventricular injection of FGF1 at low doses sustains remission of hyperglycemia in obese mice and diabetic rats (Scarlett et al., 2016). FGF1’s central effect in lowering hyperglycemia occurs through the induction of hepatic glucokinase, which increases hepatic glucose uptake (Scarlett et al., 2019). However, the specific neuronal types and brain regions involved in FGF1’s central anti-hyperglycemia actions remain unidentified.

Although FGF1 has antidiabetic effects, the tumorigenic risks associated with chronic FGF1 administration have raised concerns about its use as a pharmacotherapy for diabetes (Jiao et al., 2015; Kwabi-Addo et al., 2004). However, the mitogenic and antidiabetic activities of FGF1 appear to be separable. FGF1 induces cell proliferation mainly via FGFR3 and FGFR4, while its metabolic activity is predominantly mediated by FGFR1 (Degirolamo et al., 2016; Suh et al., 2014; Wu et al., 2011). Engineered FGF1 variants, such as FGF1^ΔHBS^ (which abolishes heparan sulfate-assisted FGFR dimerization/activation) and FGF1^ΔNT^ (with 24 N-terminal amino acids deleted), have been developed to reduce its mitogenic properties while retaining metabolic activity (Huang et al., 2017; Suh et al., 2014).

### Bone morphogenic proteins

Bone morphogenic proteins (BMPs), a subset of the TGFβ superfamily of cell-regulatory proteins, are crucial for osteogenic and chondrogenic differentiation. They play pleiotropic roles in cellular differentiation, proliferation, and survival (Xiao et al., 2007). Emerging evidence suggests that BMPs have diverse functions in the development and progression of MASLD/MASH.

#### BMP2

Treatment of hepatoma cells with BMP2 induces the expression and activity of *DGAT2* (the gene involved in triglyceride synthesis) through intracellular SMAD signaling, suggesting a potential role for BMP2 in hepatic lipid metabolism (Thayer et al., 2020). Palmitic acid upregulates BMP2 expression and secretion in human hepatocytes. BMP2 expression is abnormally elevated in the livers of patients with MASLD compared with normal liver tissues, as reflected in higher serum BMP2 levels. An algorithm based on serum BMP2 levels and clinically relevant MASLD variables can distinguish MASH (Maranon et al., 2022), indicating that abnormal BMP2 expression is linked to MASLD/MASH.

#### BMP4

BMP4 has been shown to inhibit HFD-induced hepatic steatosis in mice by regulating genes involved in lipid metabolism and mTORC1 signaling in hepatocytes (Peng et al., 2019). Studies using spheroids composed of human stellate cells and hepatocytes demonstrate that BMP4 exhibits anti-senescent, anti-steatotic, anti-inflammatory, and anti-fibrotic effects (Baboota et al., 2022). BMP4 suppresses markers of hepatic steatosis, inflammation, and liver injury by upregulating glutathione peroxidase 4 (GPX4), thereby reducing ferroptosis (Wang et al., 2022a). Moreover, BMP4 expression is upregulated in both patients and mice with MASLD (Peng et al., 2019; Wang et al., 2022a).

#### BMP6

Similar to BMP4, BMP6 appears to protect against MASLD/MASH. BMP6 is upregulated in MASLD and is correlated with hepatic steatosis but not with liver inflammation or injury (Arndt et al., 2015). Interestingly, BMP6 is solely elevated in MASLD but not in other murine liver injury models or diseased human livers (Arndt et al., 2015). Compared with wild-type mice, BMP6-deficient mice exhibit increased hepatic inflammation and fibrosis following methionine-choline-deficient (MCD) and HFD feeding (Arndt et al., 2015). Recombinant BMP6 inhibits HSCs activation and reduces proinflammatory and profibrogenic gene expression in activated HSCs (Arndt et al., 2015). Thus, we speculate that upregulation of BMP6 in MASLD may be hepatoprotective, providing a promising anti-steatotic and anti-fibrogenic strategy.

#### BMP7

BMP7 has been demonstrated to exert therapeutic potential for MASLD. Treating HFD-induced obese mice and *ob*/*ob* mice with liver-directed adeno-associated virus (AAV)-BMP7 vectors increases circulating BMP7 levels, induces the browning of WAT, activates BAT, alleviates hepatic steatosis, and normalizes body weight and insulin resistance (Casana et al., 2022), highlights the potential of AAV-BMP7-mediated gene therapy for the treatment of MASLD/MASH.

#### BMP8B

In contrast, BMP8B seems to be positively associated with MASLD/MASH progression. BMP8B has been shown to promote fatty acid uptake, *de novo* lipogenesis, NF-κB activation, and proinflammatory gene expression in hepatocytes (Mahli et al., 2019). BMP8B also promotes the proinflammatory phenotype in HSCs through both the SMAD2/3 and SMAD1/5/9 branches of the TGFβ/BMP pathway (Vacca et al., 2020). *In vivo*, the absence of BMP8B prevents HSC activation, reduces inflammation, and limits the progression of MASH (Vacca et al., 2020). Fatty acids induce BMP8B expression in a dose-dependent manner in primary human hepatocytes (Mahli et al., 2019). Furthermore, hepatic BMP8B expression is significantly increased in a murine MASLD model (Mahli et al., 2019). Notably, human data show that circulating BMP8B levels are elevated in patients with MASH compared with simple steatosis and healthy individuals. Elevated BMP8B levels are positively correlated with AST, ALT, the platelet ratio index (APRI), and the fibrosis-4 index (FIB-4) in patients with MASH (Mounika et al., 2023).

#### BMP9

The role of BMP9 in MASLD remains contentious. Some studies demonstrate its hepatoprotective activity (Yang et al., 2024). For example, BMP9-deficient mice develop hepatic steatosis due to downregulation of peroxisome proliferator-activated receptor α (PPARα) expression and reduced fatty acid oxidation. Furthermore, recombinant BMP9 treatment reduces TG accumulation in Hepa 1-6 cells (Yang et al., 2020). *In vivo* studies showed that showed that Cers6, Cidea, Fabp4 involved in lipid and glucose metabolism and Fos, Ccl2, Tlr1 involved in inflammatory response were significantly downregulated after BMP9 treatment in HFD mouse liver (Sun et al., 2020). Additionally, administration of MB109, a recombinant derivative of human BMP9, in obese mice enhances FGF21 expression, alleviates HFD-induced obesity, and reduces liver lipid droplets (Kim et al., 2016). In contrast, other studies have shown that BMP9 overexpression increases M1 macrophage gene expression (*CD86*, *IL1β*, *IL6*, *MCP-1*, *TNFα*) and the number of M1 macrophages in the liver, thereby promoting MCD diet-induced steatohepatitis in mice (Jiang et al., 2021a). Besides, Breitkopf-Heinlein et al. identified quiescent and activated HSCs as the major BMP9 producing liver cell type (Breitkopf-Heinlein et al., 2017). Upon HSC activation, endogenous BMP-9 levels increase, causing enhanced damage upon acute or chronic injury. The *in vitro* experiments showed that treatment of cultured hepatocytes with BMP9 inhibited proliferation, epithelial to mesenchymal transition and preserved expression of metabolic enzymes like cytochrome P450. As a result, *in vivo* application of BMP9 after partial hepatectomy significantly enhanced liver damage and disturbed the proliferative response (Breitkopf-Heinlein et al., 2017). Therefore, these conflicting findings suggest that further efforts should be made to investigate the function and mechanism of BMP9 in the liver.

### Emerging secreted proteins associated with MASLD

Some other secreted proteins, such as Isthmin-1 (Ism1) and mesencephalic astrocyte-derived neurotrophic factor (MANF), have been identified as regulators of MASLD/MASH. Ism1 was originally discovered in 2002 (Pera et al., 2002). As an adipokine, Ism1 plays dual roles: it enhances adipocyte glucose uptake while suppressing hepatic lipid synthesis, thereby improving hyperglycemia and reducing lipid accumulation in mouse models through the activation of PI3K-AKT signaling pathway. Notably, recombinant Ism1 alleviates hepatic steatosis by suppressing SREBP-1c in a diet-induced fatty liver mouse model and ameliorates diabetes in diet-induced obese mice. Ism1 shares glucoregulatory functions with insulin and counters liver lipid accumulation by shifting hepatocytes from a lipogenic state to a protein-synthesis state (Jiang et al., 2021b). Overall, Ism1 emerges as an intriguing secreted factor that regulates glycolipid metabolism. Future research should focus on identifying the receptors modulated by Ism1 that mediate its regulatory functions on glucose and lipid homeostasis.

MANF is an endoplasmic reticulum-resident protein first identified in 2003 (Petrova et al., 2003). *In vitro* and *in vivo* studies have demonstrated that MANF plays a role in suppressing lipogenesis by modulating SREBP-1 expression (He et al., 2020; Yan et al., 2022). Liver-specific MANF knockout mice develop hepatic steatosis, whereas MANF overexpression reduces hepatic lipid accumulation (Wu et al., 2021; Yan et al., 2022). Decreased MANF expression results in liver damage, steatosis, and fibrosis, while MANF supplementation improves diet-induced steatosis and age-related metabolic dysfunction (Sousa-Victor et al., 2019). Recent data suggest that MANF deficiency in hepatocytes or hepatic monocyte/macrophages exacerbates hepatic fibrosis. Notably, systemic administration of recombinant human MANF markedly alleviates CCl_4_-induced hepatic fibrosis in both wild-type and hepatocyte-specific MANF knockout mice by targeting TLR4/NF-κB signaling (Hou et al., 2023). Additionally, MANF inhibits HCC through suppressing NF-κB/Snail signaling (Liu et al., 2020a). Thus, MANF has shown beneficial effects in MASLD/MASH and related chronic liver diseases.

## Conclusions and future perspectives

The secretome holds significant potential for regulating liver metabolism and systemic homeostasis. Current research in this area is important for the development of novel and potentially effective treatments for MASLD/MASH. Thus, understanding the role of secreted proteins, such as hepatokines and adipokines, will enhance our understanding of hepatic steatosis, inflammation, fibrosis, cirrhosis, and HCC. For diagnostic purposes, some secreted proteins have emerged as noninvasive diagnostic markers, such as SPARCL1 and GDF3. For example, we found that the SPARCL1-ALT-AST model outperforms the ALT-AST model in identifying patients with MASH, demonstrating its value in assessing liver function. In terms of treatment, optimizing the beneficial effects of secreted proteins while minimizing risks is crucial. For instance, enhancing IL-22’s metabolic protective effects while preventing its pro-regeneration and pro-inflammation impacts on specific organs and tissues is essential. In the near future, with the development of multi-omics and more comprehensive models, such as organoid models or humanized mice, identification of secreted proteins and investigation of their roles and mechanisms will become easier, offering exciting potential as therapeutic targets for MASLD/MASH. However, further research is necessary to fully understand their functions, interactions, and to overcome the challenges of translating this knowledge into pharmacological therapies. In addition, to ensure the clinical application of secreted proteins, more issues need to be considered:

### Physiological functions and heterogeneity

Though usually neglected, understanding the physiological functions of secreted proteins is also important, including their regulatory roles in normal hepatocytes, macrophages, HSCs, as well as their expression patterns and rhythmic regulation. In addition, MASLD/MASH is a heterogeneous disease, and different subtypes may exhibit distinct patterns of dysregulation in secreted protein. Ethnicity, age, gender, and comorbidities may influence the dynamics of secreted proteins in MASLD/MASH. Future studies should investigate how these factors, along with disease severity and histological features, impact the regulation of secreted proteins.

### Upstream regulatory mechanisms

The upstream regulatory factors and mechanisms governing the expression and secretion of secreted proteins are yet to be fully elucidated. Whether and how nutrients and diets, calorie restriction, temperature, exercise, mood, air pollution, and other factors could regulate secreted proteins warrants further study.

### Systemic and adverse effects

Some secreted proteins may have systemic or even adverse effects. For instance, FGF21 has been shown to induce severe bone loss, osteoporosis, and affect fertility in mice (Liu et al., 2024; Moeckli et al., 2022; Wei et al., 2012). Thus, hepatocytes-specific delivery strategies, such as utilizing lipid nanoparticle (LNP) or N-Acetylgalactosamine (GalNAC), could be employed to specifically target secreted protein in treating MASLD/MASH while minimizing side effects.

### Translational challenges

Differences in metabolism and physiology between animal models and humans often result in divergent responses to therapies. For instance, while GDF15 treatment shows significant weight loss effects in preclinical studies, its efficacy in humans is limited. To address this challenge, it is essential to prioritize the development of more clinically relevant animal models. Advanced technologies, such as humanized animal models, 3D organoids, and microphysiological systems, can better replicate human diseases and enhance the predictability of therapeutic outcomes.

## Data Availability

Not applicable.
